# Linking metabolomics data to underlying metabolic regulation

**DOI:** 10.3389/fmolb.2014.00022

**Published:** 2014-11-06

**Authors:** Thomas Nägele

**Affiliations:** Department of Ecogenomics and Systems Biology, University of ViennaVienna, Austria

**Keywords:** metabolomics, GC-MS, LC-MS, systems biology, metabolic regulation, cellular compartmentalization, multivariate statistics, mathematical modeling

## Abstract

The comprehensive experimental analysis of a metabolic constitution plays a central role in approaches of organismal systems biology. Quantifying the impact of a changing environment on the homeostasis of cellular metabolism has been the focus of numerous studies applying various metabolomics techniques. It has been proven that approaches which integrate different analytical techniques, e.g., LC-MS, GC-MS, CE-MS and H-NMR, can provide a comprehensive picture of a certain metabolic homeostasis. Identification of metabolic compounds and quantification of metabolite levels represent the groundwork for the analysis of regulatory strategies in cellular metabolism. This significantly promotes our current understanding of the molecular organization and regulation of cells, tissues and whole organisms. Nevertheless, it is demanding to elicit the pertinent information which is contained in metabolomics data sets. Based on the central dogma of molecular biology, metabolite levels and their fluctuations are the result of a directed flux of information from gene activation over transcription to translation and posttranslational modification. Hence, metabolomics data represent the summed output of a metabolic system comprising various levels of molecular organization. As a consequence, the inverse assignment of metabolomics data to underlying regulatory processes should yield information which—if deciphered correctly—provides comprehensive insight into a metabolic system. Yet, the deduction of regulatory principles is complex not only due to the high number of metabolic compounds, but also because of a high level of cellular compartmentalization and differentiation. Motivated by the question how metabolomics approaches can provide a representative view on regulatory biochemical processes, this article intends to present and discuss current metabolomics applications, strategies of data analysis and their limitations with respect to the interpretability in context of biological processes.

## Introduction

Systems biology has become a rapidly growing research field aiming at a comprehensive representation of complex biological systems. Metabolomics plays a central role in systems biology as it provides essential information about the metabolome, i.e., the metabolic constitution and the dynamic behavior of metabolite levels. The combination of chromatographic techniques and mass spectrometry detection has enabled the rapid and precise high-throughput analysis of up to hundreds, or even thousands, of metabolic compounds from the same sample (Hall et al., 2002). Yet, the full scientific potential of metabolomics techniques is still limited due to a considerable variation in annotation confidence (Creek et al., 2014). As discussed by Creek and co-workers, one possibility to gain relatively high confidence is the comparison of multiple physicochemical properties of an authentic pure chemical standard to those of the metabolite of interest. Techniques like the comprehensive GCxGC—*time of flight mass spectrometry* (GCxGC-TOFMS), where two columns with orthogonal separation characteristics are combined, yield a much higher peak capacity (Almstetter et al., 2012) and may help increase the identification confidence by resolving co-eluting compounds. But also on the level of mass spectrometry such co-elutions might be resolved, for instance by applying techniques of tandem-MS or MS^n^ (Mei-Ling et al., 2006). The resulting data matrix, which typically comprises compounds of the central carbon/nitrogen metabolism, i.e., sugars, amino acids, organic acids and lipids, characterizes a metabolic homeostasis or its perturbation-induced dynamics (Kaplan et al., 2004; Leon et al., 2013; Aldridge and Rhee, 2014). Chemical derivatization broadens the spectrum of metabolites which can be assessed by GC techniques making them become volatile and thermally stable (Roessner et al., 2000). A commonly used method is a two-step derivatization comprising oximation and silylation. While the in oximation step sugars are stabilized in an open ring conformation, the silylation step stabilizes molecules by replacing hydrogen in functional polar groups, e.g., the hydroxyl group, by a trimethylsilyl group [-Si(CH_3_)_3_] (Hill and Roessner, 2013). While GC-MS particularly enables the quantification of volatile and uncharged compounds, Liquid chromatography coupled to mass spectrometry (LC-MS) is the method of choice to analyse semi- or non-volatile and thermally unstable compounds (Hopfgartner and Varesio, 2013). Both GC-MS and LC-MS might be applied to analyse isomeric compounds, but the high chromatographic resolution of GC, and particularly GCxGC, make it a suitable analytical technique to resolve structurally closely related compounds (Meinert and Meierhenrich, 2012). In LC-MS, structural information about molecules can be obtained by collision-induced dissociation (Jennings, 2000). Different techniques in mass spectrometry as well as their characteristic features are summarized elsewhere (e.g., see Weckwerth, 2011), but it can be generalized that the combination of those techniques is expected to increase the coverage of a metabolome significantly.

Despite identification confidence clearly being the limiting factor, the output of a GC- and LC-MS platform yields a comprehensive view of a metabolic homeostasis and its response to genomic or environmental perturbation. Beyond, there are further analytical techniques, such as UV, IR, FT-IR, and FT-ICR spectroscopy (Van Agthoven et al., 2013), nuclear magnetic resonance (NMR) (Simmler et al., 2014) or capillary electrophoresis (Kuehnbaum and Britz-Mckibbin, 2013), which can even enlarge this metabolic information space and increase its confidence. The metabolic coverage yielded by metabolomics approaches depends on the organism and sample type being analyzed. While the prokaryotic metabolome of *E. coli* comprises about 750 metabolites (Nobeli et al., 2003), eukaryotic metabolomes have been described to range from more than 1000 (e.g., yeast Herrgard et al., 2008) over several thousands (e.g., humans Duarte et al., 2007) up to tens or even hundreds of thousands in plants (Hall et al., 2002). Particularly in eukaryotic organisms, the interpretation of metabolomics data is complex not only due to the high number of metabolic compounds but also because of a high level of cellular compartmentalization, different cell types, tissues and organs. In a metabolomics study on the alga *Chara australis* the subcellular localization and dynamics of 125 metabolites was analyzed revealing a stress-induced asynchronous fluctuation of metabolite levels in the vacuole and cytosol (Oikawa et al., 2011). Based on their findings the authors suggested that metabolite levels are regulated separately in intracellular compartments. Also in higher plants, several studies have focused the subcellular analysis of metabolite dynamics and underlying fluxes (Masakapalli et al., 2010, 2013; Klie et al., 2011; Krueger et al., 2011; Nägele and Heyer, 2013; Szecowka et al., 2013; Arrivault et al., 2014). As it is a characteristic feature of eukaryotic cells, the experimental analysis of subcellular organization of metabolism cannot be over assessed. Due to the high information content of studies resolving the subcellular level, it is possible to unravel unexpected features of metabolic regulation. A concrete example for the need of subcellular resolution is the existence of plastidial and cytosolic pathways for carbohydrate oxidation, i.e., glycolysis or the oxidative pentose phosphate pathway (PPP). In a study of subcellular flux analysis in a heterotrophic Arabidopsis cell suspension using steady-state stable isotope labeling, it has been shown that multiple data sets can be fitted successfully to models with an altered subcellular compartmentation of the PPP (Masakapalli et al., 2010). With their approach, the authors provide evidence for the importance of experimental data on the subcellular level in order to reduce the uncertainty about the interpretation of biochemical regulatory processes. Another comprehensive example for subcellular analysis of leaf metabolism in Arabidopsis was provided recently by using a strategy of ^13^CO_2_-labeling to resolve time-dependent patterns and kinetics in the metabolome (Szecowka et al., 2013). Changes in isotope patterns of 40 primary metabolites were analyzed using LC- or GC-MS comprising central carbohydrates, organic, and amino acids as well as phosphorylated intermediates, e.g., from the Calvin-Benson cycle. While many of the experimental findings were according to previous expectations, several unexpected features of labeling kinetics could be unraveled due to the subcellular resolution (Szecowka et al., 2013). Finally, the authors conclude that information about metabolite compartmentation is a prerequisite for modeling photosynthetic and/or other metabolic processes in multicellular eukaryotic tissues.

The above mentioned studies present reasonable and comprehensive approaches to assess complex systems in a proportionally detailed manner. However, there exists a clear discrepancy between the number of metabolites which are absolutely quantified (~10–100) or identified and (relatively) quantified (~100–1000), and which are expected to be found in a metabolome based on genome-wide predictions (~10^3^–10^5^). Beyond the objective of increasing the coverage and confidence of analytical metabolomics platforms it is a particular challenge to interpret the resulting experimental information in a biochemical meaningful way which is the prerequisite for the successful generation of a testable hypothesis. As outlined above, this is mainly due to the high information content of underlying molecular and structural organization being compiled in a metabolomics data set. In this context, the following chapter strives to outline open questions and different data evaluation strategies applied and developed in the metabolomics research field.

## Deriving regulatory strategies from metabolic snapshots

The difficulty of interpreting comprehensive metabolomics data sets can easily be retraced by a simple example: an experiment comprising samples of two groups, e.g., a wild type (control group) and a *knock out* mutant (treatment group), typically results in *m* independent biological replicates each with *n* technical replicates for each group. Let *p* denote the number of metabolites being quantified in the metabolomics experiment. Then the summary of all experimental data within this experimental design ends up in two data arrays each with *p* × *n* × *m* dimensions. Assuming that technical variance of the method or platform has been shown to be much lower than biological variance (var_tech_ << var_biol_), a technical mean value can be built for each biological replicate and the data arrays reduce to data matrices with *p* × *m* dimensions. Although “basic” univariate statistical methods and tests immediately allow for a direct comparison of different data sets and provide a first idea of underlying biological mechanisms, generated hypotheses frequently suffer from ambiguity due to various biochemical explanations for one metabolomic feature. The covariance matrix of a metabolomics data set helps to quantify such an ambiguity. It is a statistical measure for how components, i.e., metabolites, are related to each other (Equation 1).

(1) $$Cov(x,y)=\frac{1}{m-1}\sum_{i=1}^{m}\left(x_{i}-\bar{x}\right)(y_{i}-\bar{y})$$

*x* and *y* denote variables (*here:* metabolite levels) with a sample size *m* and a mean value *x* and *y*. Accordingly, a *p* × *p* covariance matrix is derived from a *p* × *m* data matrix. This quadratic relationship between the number of metabolites and possible (metabolic) interactions exemplarily demonstrates why even small metabolomics data sets, i.e., *p* < 10, are difficult to interpret without appropriate mathematical methods: increasing the metabolomic coverage of experimental approaches is automatically linked to an exponential increase in possible explanations which have to be considered. Thus, to find the most reasonable and comprehensive biochemical explanation for all resolved experimental data, the simultaneous application of various statistical methods which confirm or even complement each other has become a common approach. Basic statistical methods are well-established and numerous platforms and graphical user interfaces (GUIs) have been developed to enable and facilitate the application of such methods [for an overview of available tools and software packages see e.g., (Sugimoto et al., 2012) or (Sheth and Thaker, 2014)]. Most of these statistical platforms even go beyond and are capable of analysing, resolving and visualizing multivariate problems, applying methods like the principal component analysis (PCA), the independent component analysis (ICA) (Steuer et al., 2007), or even the independent PCA (Yao et al., 2012). Based on the (co-)variance information, these multivariate techniques reduce the dimensionality of the dataset while retaining as much as information, i.e., variance, as possible. This yields abstract variables, i.e., components, which imply the most pronounced and characteristic features of multivariate data sets, thus enabling and facilitating the generation of biological hypotheses. Complemented with other unsupervised and supervised statistical methods, e.g., hierarchical clustering, *k*-means clustering and partial least squares discriminant analysis (PLS-DA), molecular compounds contributing to the separation of the samples by a varying degree, can be identified (Okada et al., 2010; Westerhuis et al., 2010; Le Cao et al., 2011; Korman et al., 2012; Sun and Weckwerth, 2012; Bellaire et al., 2014; Madala et al., 2014; Uarrota et al., 2014).

**Figure 1:**
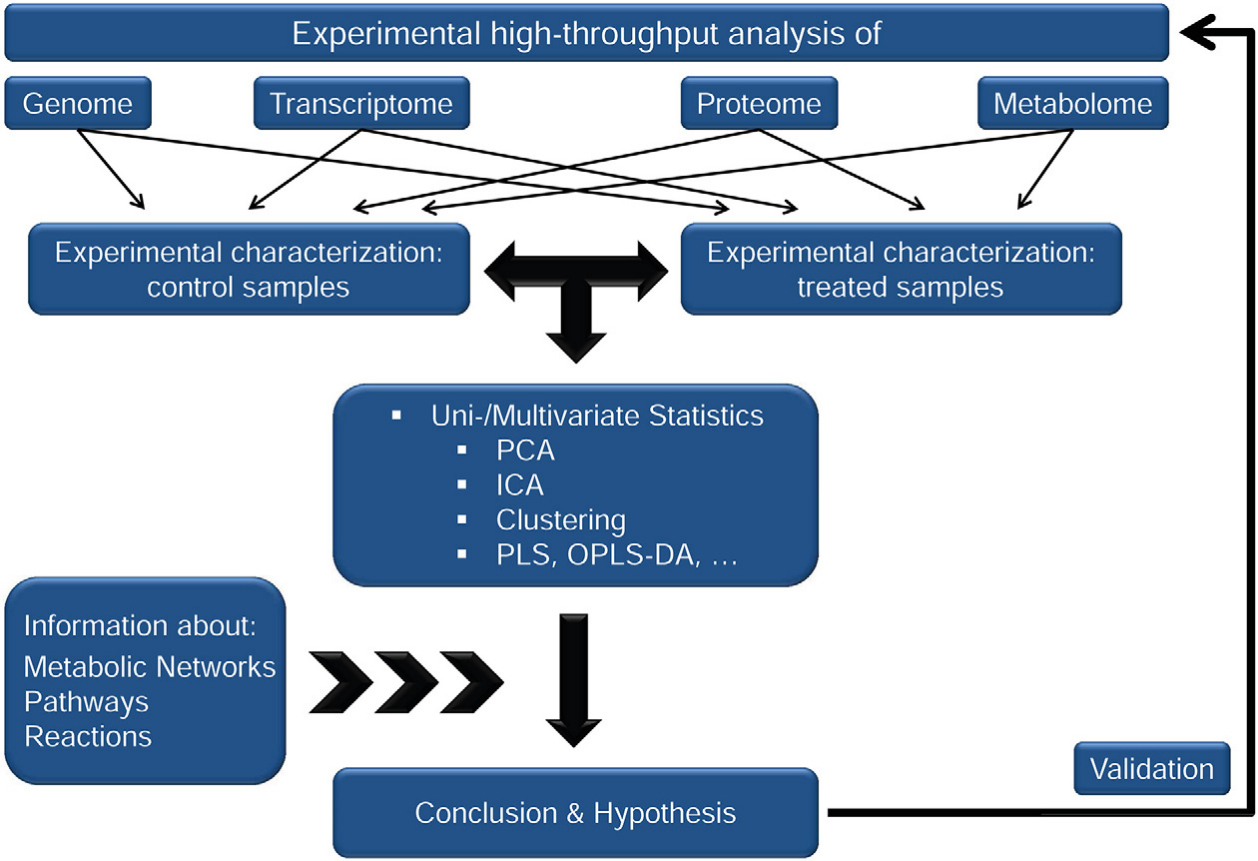
**A typical systems biology workflow to derive and test hypotheses from experimental high-throughput analysis**.

The abovementioned strategies of (multivariate) data analysis are an integral part of most systems biology approaches comprising not only metabolomics but, mostly, also other data sets derived from various “omics” techniques, for example transcriptomics and proteomics (Weckwerth, 2008). Special software packages have been developed for the integrative analysis of various experimental high-throughput data sets aiming at a comprehensive regression analysis, correlation and visualization. An excellent overview of software tools, platforms and workflows of computational tasks in systems biology was provided previously (Ghosh et al., 2011). Although these platforms provide a wide variety of statistical and mathematical methods, the final outcome which a system biologist is interested in may always be similar: the identification of specific metabolic clusters and patterns, i.e., samples with similar statistical characteristics, separating the control from a treatment group. This finally provides us with an idea about which metabolic steps or pathways are most likely affected by the introduced perturbation and conclusions about biochemical or molecular biological mechanisms can be drawn resulting in a testable hypothesis (Figure 1). Numerous studies from various fields of biological research have proven the applicability and usefulness of such computational workflows (Altaf-Ul-Amin et al., 2014). But contemperaneously, deriving information on the regulatory interaction between levels of molecular organization has been recognized to be accessible only to a very limited degree. This is mainly due to the complex and non-linear relationship which exists between levels of transcripts, proteins, and metabolites. Hence, if the level of a metabolite changes due to a genomic or environmental perturbation this may have various reasons: (i) feed-back/forward regulation of enzymes (with non-linear kinetics), (ii) posttranslational modification of enzymes/proteins involved in the metabolite synthesis, interconversion or transport, (iii) changes in the rate of protein biosynthesis and/or degradation, and so on. As a consequence, drawing an unambiguous conclusion directly from a set of metabolite levels on regulatory strategies in a metabolic network is hardly possible—except for the particular case that (A) all components of the metabolic network are known and biochemically characterized, or (B) all relationships between levels of molecular organization can—due to simplification—be considered as linear. Although during recent years, a lot of information about genome-wide metabolic network structures and regulatory principles has been unraveled, comprising, to name only a few, molecular systems of prokaryotes (Carrera et al., 2014), yeast (Sanchez et al., 2014), algae (Chang et al., 2011), higher plants (Mintz-Oron et al., 2012; Hill et al., 2013), human metabolism (Mardinoglu et al., 2013), and disease and medicine (Vandamme et al., 2013), we are still far away from being able to fulfill all—or at least most of the—requirements being necessary for approach (A). In contrast, strategies of mathematical modeling enable the procedure of linearization which is explained and discussed in the following paragraph.

## Linearization of metabolic functions

The linearization of non-linear functions around a certain state is a frequently applied approach in mathematics, physics or control theory to approximate solutions of complex and chaotic systems (Strogatz, 1994). In a very figurative context one could think of a polynomial function *y*(*x*), containing several minima and maxima. Yet, instead of considering and analysing the function *y*(*x*) for all *x*, we are only interested in the behavior of *y*(*x*) in one certain point *x*_0_. In this point, the function is described by the coordinates (*x*_0_, *y*(*x*_0_)). We can approximate the function *y*(*x*) by drawing the tangent in (*x*_0_, *y*(*x*_0_). With this, we have approximated the original function by a linear function. In a metabolic context, *x*_0_ would represent a certain steady-state metabolite concentration while *y*(*x*) represents the so-called metabolite function taking the steady-state value *y*(*x*_0_). In the following paragraphs, the time-dependent metabolic function will be re-written as *f_M_*(*t*).

Comparing the linearized approximation with the original function it becomes obvious that these solutions are only valid in a very narrow and predefined interval of the fundamental function. However, they still provide a highly informative insight and can help to trace back basic principles of the original function—particularly if this function is highly complex. Ultimately, they may allow for the simulation and prediction of the systems behavior, thereby broadening the current knowledge about the origin of the system's complexity.

**Figure 2:**
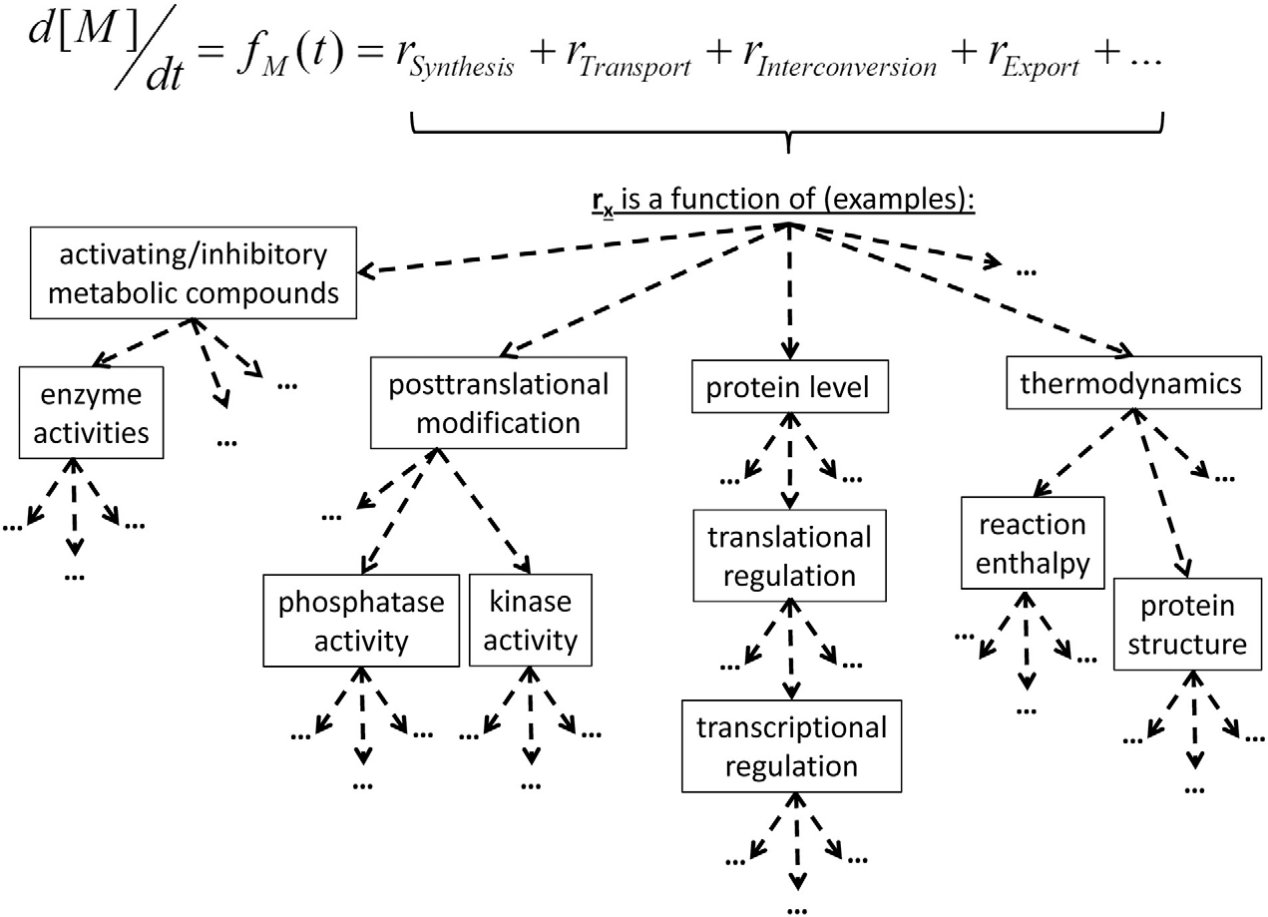
**Definition of metabolic functions, *f_M_*(*t*), and examples for regulatory processes**. The dashed arrows indicate functional dependencies and can be read like “is a function of.”

**Figure 3:**
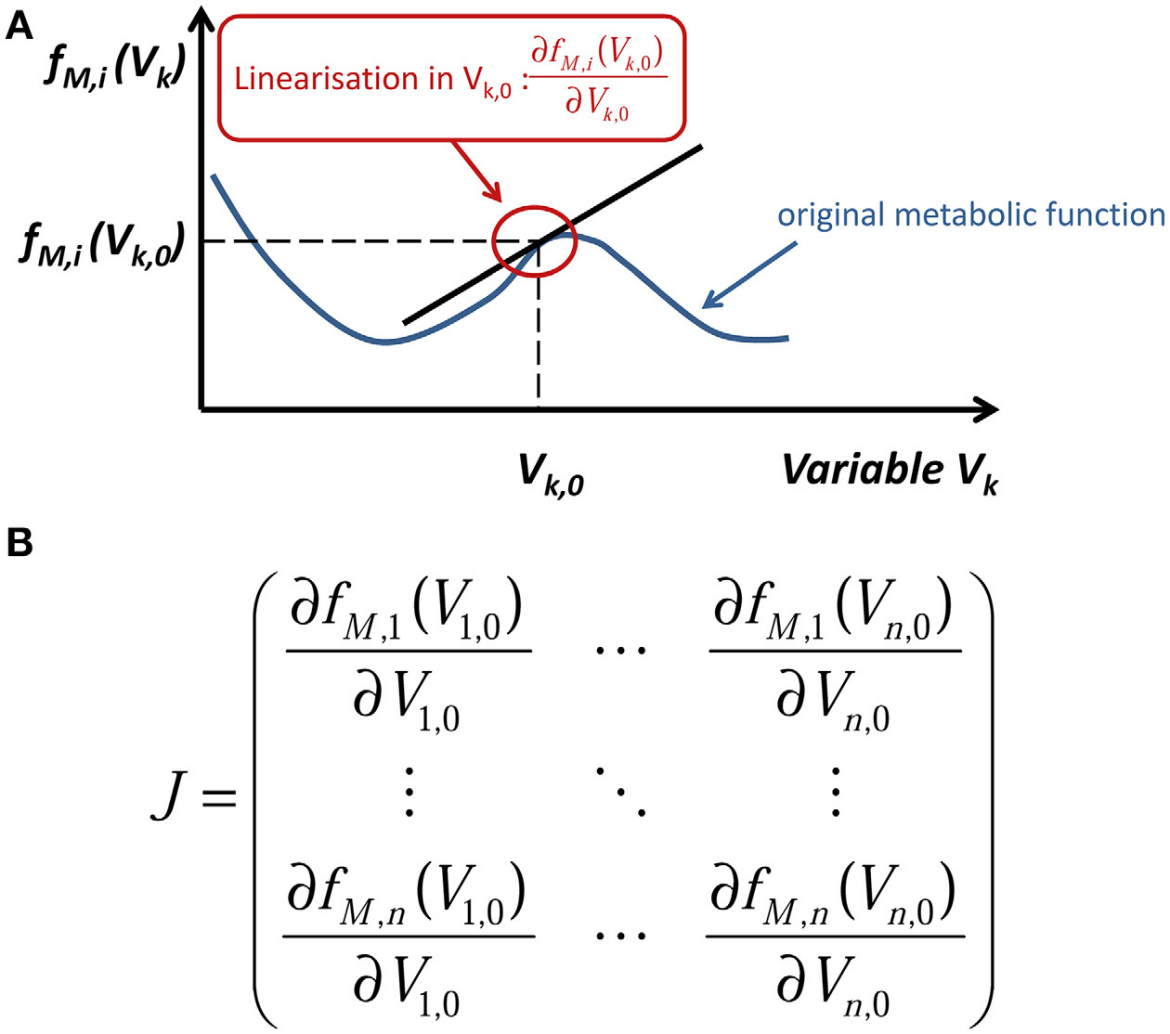
**Schematic representation of the linearization process of a metabolic function. (A)** Graphical draft of the linearization procedure of a metabolic function, *f_M,i_*(*V_k_*), at a certain metabolic state, *V*_*k*,0_. **(B)** The Jacobian matrix *J* comprising all results of the linearization process, i.e., partial derivatives.

To exemplarily transfer this strategy to a systems biology approach, we consider a time-dependent change in the concentration of a metabolite *M*. Mathematically this can be expressed by an ordinary differential equation (ODE). While the left side of the equation describes the changes in *M* with changes in time *t*, the right side of the equation describes all (reaction) rates affecting the concentration of *M*. This can be summarized by the metabolic function *f_M_*(*t*). The reaction rates again depend on various parameters, variables and functions, such as inhibitor/activator concentrations, thermodynamic constraints, posttranslational modification, protein levels, and so on. This automatically connects the regulation of metabolite levels to all other levels of molecular organization (Figure 2). Each of the functions contains various non-linear elements, e.g., enzyme kinetics (Michaelis-Menten/Hill/…) or thermodynamic equations (Arrhenius/…), resulting in a highly non-linear description of the biological system. Applying the principle of linearization allows for the characterization of such a non-linear system around an experimentally analyzed metabolic (steady) state by replacing the non-linear with linear functions. In a simple two-dimensional example this can be illustrated by the tangent in one point of the original metabolic function (Figure 3A). Mathematically this is performed by differentiating the metabolic function with respect to a reaction variable, such as time, metabolites or other parts of the abovementioned molecular organization (see Figure 2). If this is performed for all considered system variables at the same metabolic steady-state, the partial derivatives are combined in the so-called Jacobian matrix (Figure 3B). By inducing perturbations and analysing the deflection of the metabolic system, characteristic features, like stability of stead-states or sensitivity of fluxes, can be estimated and summarized in the Jacobian matrix (Steuer et al., 2006; Chen and Chen, 2009; Reznik and Segre, 2010). While the mathematical theory behind these approaches is fully established (Strogatz, 1994) and is commonly applied in engineering sciences (Föllinger and Konigorski, 2013), the direct integration of experimental (metabolomics) data is still challenging. In recent work, we have focused on the development of methods for a direct integration of metabolomics data, merging the covariance information with a genome-wide metabolic network structure. Thereby, we could successfully link experimental metabolomics data to strategies of subcellular compartmentation (Nägele and Weckwerth, 2013) and regulation of enzyme activity (Nägele et al., 2014). Listing all the presented approaches together with many others from the research fields of biomathematics, theoretical biology, bioinformatics and cybernetics, will yield an astonishing diversity of comprehensive models and strategies, which have been developed during the last decades. One central challenge of the next decades' metabolomics research will be the integration and application of many of these theoretical platforms, to exploit the experimental high-throughput data as efficient as possible.

## Conclusion and outlook

Summarizing the above mentioned findings, open questions and approaches, metabolomics plays a central role in current systems biology research. Future work on the integration of different experimental metabolomics techniques will broaden the coverage of metabolomes. The interpretation of resulting multidimensional data arrays in context of metabolic network information at genome-scale will significantly promote our understanding of complex metabolic networks. Combining and integrating statistical methods and strategies of mathematical modeling is a promising approach to improve our skills in construing comprehensive metabolomics data with respect to biochemical regulation in multi-layered and highly compartmentalized biological systems. This will essentially contribute to a very profound knowledge across all domains of life and provide us with new ideas and perspectives to solve upcoming questions of global concern.

### Conflict of interest statement

The author declares that the research was conducted in the absence of any commercial or financial relationships that could be construed as a potential conflict of interest.
